# Adhesion Molecules ICAM-1 and PECAM-1 as Potential Biomarkers of Central Nervous System Damage in Women Breast Cancer Survivors

**DOI:** 10.3390/pathophysiology29010006

**Published:** 2022-02-16

**Authors:** Maria Pospelova, Varvara Krasnikova, Olga Fionik, Tatyana Alekseeva, Konstantin Samochernykh, Nataliya Ivanova, Nikita Trofimov, Tatyana Vavilova, Elena Vasilieva, Mariya Topuzova, Alexandra Chaykovskaya, Albina Makhanova, Tatyana Bukkieva, Evgeniya Kayumova, Stephanie Combs, Maxim Shevtsov

**Affiliations:** 1 Personalized Medicine Centre, Almazov National Medical Research Centre, 2 Akkuratova Str., 197341 Saint Petersburg, Russia; pospelovaml@mail.ru (M.P.); varya.krasnikova.93@mail.ru (V.K.); fvolga@mail.ru (O.F.); atmspb@mail.ru (T.A.); neurobaby12@gmail.com (K.S.); ivamel@yandex.ru (N.I.); nikita.trofimov.1999@mail.ru (N.T.); vtv.lab.spb@gmail.com (T.V.); elena-almazlab@yandex.ru (E.V.); topuzova_mp@almazovcentre.ru (M.T.); mumu6394@gmail.com (A.C.); a.mahanova.a@mail.ru (A.M.); tanya-book25@mail.ru (T.B.); kazqwer@yandex.ru (E.K.); 2 Department of Radiation Oncology, Technishe Universität München (TUM), Klinikum Rechts der Isar, Ismaninger Str. 22, 81675 Munich, Germany; stephanie.combs@tum.de; 3 Laboratory of Biomedical Nanotechnologies, Institute of Cytology of the Russian Academy of Sciences (RAS), Tikhoretsky Ave., 4, 194064 Saint Petersburg, Russia; 4 Laboratory of Biomedical Cell Technologies, Far Eastern Federal University, 690091 Vladivostok, Russia

**Keywords:** breast cancer survivors, post-mastectomy pain syndrome, breast cancer, adhesion molecules, central nervous system damage, PECAM-1, ICAM-1, liquid biopsy

## Abstract

Breast cancer (BC) is the most common tumor in women worldwide with high mortality rates. Surgical methods followed by radio–chemotherapy are used to treat these tumors. Such treatment can lead to various side effects, including neurological complications. The development of a reliable biomarker to predict the onset of CNS complications could improve clinical outcomes. In the current study, ICAM-1 and PECAM-1 serum levels were measured as potential biomarkers in 45 female patients in a long-term follow-up period after breast cancer treatment, and compared to 25 age-matched female healthy volunteers. Serum levels of both biomarkers, ICAM-1 and PECAM-1 were significantly higher in patients after breast cancer treatment and could be associated with cognitive dysfunction, depression, and vestibulocerebellar ataxia. In conclusion, our results provide a first hint that elevated serum levels of ICAM-1 and PECAM-1 could serve as early predictive biomarkers in breast cancer survivors that might help to improve the management of these patients.

## 1. Introduction

According to the GLOBOCAN study, which estimates the prevalence of cancer in 185 countries around the world, breast cancer ranks first in malignant tumors in women. In 2018, 2.1 million women were diagnosed with breast cancer worldwide [1]. In Russia, the incidence of breast cancer constitutes 65.5 cases per 100,000 women [2] and surgical treatment of breast cancer is carried out in 95.9% of these patients. In 71%, surgery is combined with additional treatment modalities such as radio- and/or chemotherapy. The progressive development of a number of organic and functional complications after the treatment of breast cancer results in long-term side effects in more than 80% of the patients [3].

It has been proven that in 24–90% of cases, after surgery in combination with radio–chemotherapy, a complex of complications develop, which can include: (i) the entrapping of nerves in the fibrotic-scar tissue which is related to surgery and ionizing radiation; (ii) hemolymphomicrocirculation disorders; and (iii) neuro-physiological disorders of the peripheral and central nervous system (CNS) [4].

In patients following breast carcinoma treatment, apart from postmastectomy pain syndrome (PMPS), clinical manifestations include secondary upper limb lymphedema (breast cancer-related lymphedema), peripheral polyneuropathy caused by chemotherapy (chemotherapy-induced polyneuropathy), and neurological manifestations (e.g., depression). The direct cytotoxic effects of the applied cytostatic drugs to neurons and glia, which change the levels of the body’s neurotransmitters and increase production of pro-inflammatory cytokines (e.g., IL-6, IL-8), could be responsible for the damage to the CNS [5]. However, the molecular mechanisms underlying these disorders are still unclear. Furthermore, predictors and reliable prognostic markers indicative for the development of various clinical manifestations of complications following breast cancer treatment have not yet been identified, and therefore, preventive and rehabilitative measures are insufficiently effective.

A promising approach could be based on the assessment of biomarkers in liquid biopsies that could potentially reflect the stages and severity of the disease manifestation. Among numerous suggested biomarkers, intercellular adhesion molecules type 1 (ICAM-1) and platelet and endothelial adhesion molecules type 1 (PECAM-1) are of particular interest. Intercellular adhesion molecule type 1 (ICAM-1) is a transmembrane glycoprotein belonging to the immunoglobulin family that is expressed on the surface of endothelial cells, neutrophils, monocytes, and lymphocytes, as well as on microglial cells and astrocytes in the central nervous system [6]. The main function of ICAM-1 is the adhesion of lymphocytes to the endothelium during their migration to the focus of inflammation [7]. Platelet and endothelial adhesion molecule type 1 (PECAM-1) belongs to the type I transmembrane glycoprotein superfamily and immunoglobulin superfamily. PECAM-1 is expressed on vascular endothelium, monocytes, polymorphonuclear neutrophils, intact T cells, and platelets. PECAM-1 is predominantly involved in the transendothelial migration of white blood cells [8,9]. Previously, it was demonstrated that ICAM-1 could be employed as a prognostic biomarker in breast cancer patients [10]. Additionally, as shown by Chen et al. ICAM-1 has a potential significance for the differential diagnosis of breast cancer and benign breast lesions [11], as well as for determining the risk of developing metastasis [12]. PECAM-1 is also involved in the process of tumor metastasis and can be used as a prognostic marker for secondary tumor lesions [13].

On the other hand, these biomarkers could be employed for clinical diagnosis of CNS non-tumor lesions. Thus, since the 1990s, an increase in ICAM-1 and PECAM-1 has been reported in patients with vascular diseases including symptomatic and asymptomatic atherosclerotic vascular lesions [9,14]. In addition, preclinical studies have shown the increased expression of ICAM-1 and PECAM-1 in irradiated heart and lung endothelial cells [15]. This could explain the increased risk of developing radiation-induced lung and heart diseases in patients who receive partial lung and heart irradiation during radiotherapy [16,17]. Increased serum levels of soluble ICAM-1 and PECAM-1 are observed in a number of neuropsychiatric disorders (i.e., depression, bipolar disorder, dementia, and progressive vascular cognitive disorders) [6,18]. A positive correlation was also found between increased soluble ICAM-1 levels and the degree of damage of the white matter of the brain in cerebral small vessel diseases [19]. The detection of microstructural changes in the white matter via changes in soluble adhesion molecules can provide a promising strategy to determine the degree of CNS damage (e.g., neurodegeneration) in patients after breast cancer treatment [20].

Presently, only a few studies have reported on the biomarkers that reflect CNS damage in breast cancer survivors.

## 2. Materials and Methods

### 2.1. Experimental Design

The study was carried out in compliance with the principles of the Helsinki Declaration of the World Medical Association with the consent of the Ethics Committee of the Federal State Budgetary Institution “Almazov National Medical Research Center” of the Ministry of Health of the Russian Federation (conclusion of 31 October 2019).

#### 2.1.1. Inclusion Criteria

Women aged 25 to 50 after modified mastectomy Madden (unilateral or bilateral breast surgery) and radio–chemotherapy who developed post-treatment symptoms associated with cancer-treated breasts, but not with primary cancerous lesions, were included in the study [21,22]. Other criteria also included the ECOG performance status of 0–1, and the absence of cardiac, endocrine, rheumatic neuromuscular or musculoskeletal disorders and other tumors.

The group of healthy female volunteers included women aged 25 to 50 years, with no history of cancer or severe somatic diseases.

All women included in the study signed written informed consent.

#### 2.1.2. Exclusion Criteria

Exclusion criteria included: signs of progression of the main oncological disease; the presence of distant metastases of breast cancer including CNS damage, the presence of protrusions and/or hernias of the intervertebral discs of the spine, ankylosing spondylitis, pathological fractures of the vertebral bodies, acute spinal injuries, conditions after spinal surgery; the presence of hemodynamically significant atherosclerotic stenoses of the head and neck main arteries; acute infectious and mental diseases, as well as other conditions that prevent neurological examination and manual diagnosis; pregnancy; decompensated somatic pathology; contraindications to MRI.

### 2.2. Clinical and Neuropsychological Assessment

Clinical and neuropsychological assessment included: assessment of complaints; anamnesis; evaluation of QoL (the Medical Outcomes Study Short Form 36-Item Health Survey (SF-36) and quality-of-life questionnaire for cancer patients, European Organization for Research and Treatment of Cancer Quality of Life Questionnaire—Core 30 (EORTC QLQ-C30); Zung depression scale; disability of the arm, shoulder and hand outcome measure (DASH); neurological examination; measurement of the volume of the upper extremities; and joints movements.

At the initial examination, complaints were collected from patients after breast cancer treatment.

The anamnesis included the period after the operation, the type of operation, the course of chemotherapy, the course of radiation therapy, the presence of relapses, and the hormonotherapy with Tamoxifen^®^.

We completed a quality-of-life assessment using the SF-36 Quality-of-Life questionnaire. The short general health status assessment questionnaire (The Medical Outcomes Study Short Form 36-Item Health Survey—SF-36) is designed to determine the degree of satisfaction of the patient with their physical, mental and social functioning in the conditions of the disease. The 36 items in the questionnaire are grouped into eight scales: physical functioning; role-playing activity; body pain; general health; vitality; social functioning; emotional state; and mental health. The indicators of each scale vary between 0 and 100, where 100 represents total health, and all scales form two indicators: mental and physical well-being. The results are presented in the form of scores in points on 8 scales, compiled in such a way that a higher score indicates a higher level of quality of life [23].

The EORTC QLQ-C30 questionnaire includes 30 questions and consists of 5 functional scales. All scales are measured in the range from 0 to 100. A high score on the functional scales represents a higher and healthier level of functioning, a high score for general health represents a high level of quality of life. A high score for the symptom scale represents a high level of symptomatology [24].

The level of depression was assessed by employing the Zung depression test. The test takes into account 20 factors that determine the four levels of depression and contains ten positively formulated and ten negatively formulated questions. Each question is rated on a scale of 1 to 4 (based on these answers: “never”, “sometimes”, “often”, “constantly”). The results are divided into four ranges: 25–49—normal range; 50–59—mildly depressed; 60–69—moderately depressed; 70 and above—severely depressed.

The function of the upper limbs was evaluated by the DASH questionnaire, consisting of 30 questions related to the state of hand function over the past week. Twenty-one of them reveal the degree of difficulty in performing various physical actions due to limited shoulder or hand function; 6 points relate to the severity of certain symptoms and 3 to social and role functions. Each item has 5 answer options, rated in points from 1 to 5. The sum of the points for all items is then converted to a 100-point scale, used to evaluate upper limb incapacity from 0 (no incapacity: good functionality) to 100 (excessive incapacity)**.**

Subjective examination included: neurological examination; measurement of the volume of the upper extremities; joints movements. During the neurological examination, the assessment of coordination tests was performed (i.e., finger–nasal test, Romberg test), and symptoms of polyneuropathy were assessed (including hypesthesia, hyperesthesia, paresthesia in the distal extremities).

Examinations included assessment of sensory perception (hypoesthesia, normal perception, hyperesthesia) in the extremities. The testing procedure was described and demonstrated to the patients. The patients were instructed to close their eyes and concentrate on the sensations evoked by stimuli administered by a physician. Test results were blinded for participants during the test procedure. First, tests were performed on the distal extremities, then on the proximal extremities on the same side. If sensitivity was impaired in the distal parts of the extremities, the presence of polyneuropathy was assumed.

The assessment of the movements in the shoulder joint on the side of the operation was performed using a goniometer and compared with the movement on the contralateral side.

The Romberg test was used to detect statistical ataxia. A finger–nose test was performed to detect dynamic ataxia. With positive results of coordination tests, vestibulo-atactic syndrome was diagnosed.

The upper extremities were measured on both sides to assess the volume of the limbs and subsequently to assess the degree of edema.

The classification based on determining the difference in the volume of an edematous limb compared to a healthy limb describes four degrees of edema: 0—subclinical condition; I—an increase in the circumference of the affected limb by less than 20%; stage II—an increase of 21–40%; stage III—an increase of more than 40% [25].

### 2.3. Analysis of Soluble Adhesion Molecules

The serum (of 7 mL blood) was collected from oncological patients’ and healthy volunteers’ blood, aliquoted, and stored at −70 °C. Assessment of soluble endothelial platelet adhesion molecule 1 (sPECAM-1) and soluble intercellular adhesion molecule 1 (sICAM-1) was performed using the commercially available Human sPECAM-1 ELISA kit and Human sICAM-1 ELISA kit (both Bender MedSystems GmbH, Germany) according to the manufacturer’s protocol.

### 2.4. Statistical Analysis

Statistical processing of the obtained data was carried out using the IBM SPSS Statistics 28.0.1.0 program. All available data were analyzed statistically. To assess the qualitative variables, absolute and relative indicators (% of the number of observations) were used. Quantitative variables were characterized by medians and ranges of values (Me [25 Percentile; 75 Percentile]). Statistical comparison of quantitative indicators was carried out using nonparametric methods. The statistical significance of changes in quantitative indicators was checked using the Mann–Whitney U test. Patients after breast cancer treatment were divided into subgroups according to the following characteristics: the presence of vestibulo-atactic syndrome, depression, polyneuropathy, lymphedema, histological types of breast cancer and hormone receptor status, as well as anamnesis of radiation therapy and chemotherapy. Statistical comparisons of quantitative variables between the two parallel subgroups were made using the Mann–Whitney U-test. Statistical comparisons of quantitative variables between the three subgroups were made using the Kruskal–Wallis H-test. The *p*-value of <0.05 was considered statistically significant. The probability of a type I error (two-sided significance level) was set at 5%.

## 3. Results

### 3.1. Clinical and Neuropsychological Evaluation of Patients

In total, 45 patients following breast cancer therapy and 25 age-matched healthy female volunteers were enrolled into the single-center controlled clinical trial. Patients and healthy women were comparable in age. All women included in the study were Caucasian. All patients were in the late postoperative period (>12 months) after radical treatment of breast cancer (Table 1).

All patients had clinical manifestations of treatment complications.

Physical examination revealed the restriction of movement in the shoulder and lymphedema of the arm.

Neurological examination revealed vestibulo-atactic syndrome and clinical manifestations of polyneuropathy (Table 2).

According to the results of the SF-36 quality-of-life questionnaire, there was a decrease in the overall physical well-being index of 40 patients (88%), and in the overall mental well-being index of 36 patients (80%). The average values in the overall physical well-being index constituted 47.04 [35.11; 58.8] and 46.24 [38; 59.2] in the general mental well-being index. The average values are presented in Table 3.

The patients were asked to complete the EORTC QLQ C30 questionnaire, where the higher (maximum 100 points) score on the functional scales represents a higher and healthier level of functioning. In group of patients after breast cancer treatment, the average value of the indicators constituted 48.24 [39.5; 52.6], which indicated a high degree of influence of the disease on QoL. The average values are presented in Table 3.

When conducting a survey of patients using the Zung depression scale, the average was 41.5 [38.4; 46.3]. If the result was more than 50 points, depression was assumed. In 18 patients (40%) signs of depression were reported. The average values are presented in Table 3.

The assessment was carried out on the DASH scale, which evaluates upper limb incapacity from 0 (no incapacity: good functionality) to 100 (excessive incapacity). The average values in the group of patients after breast cancer treatment constituted 62.97 [56.4; 66.3], indicating a pronounced violation of the function of the affected upper limb and a violation of participation in daily activity due to the restriction of movement in it. The average values are presented in Table 3.

### 3.2. PECAM-1 and ICAM-1 Serum Levels

**Figure 1:**
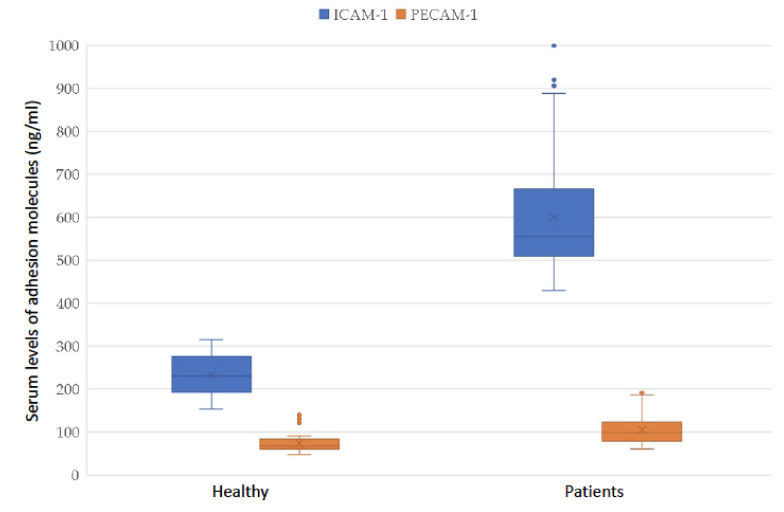
Adhesion molecules PECAM-1 and ICAM-1 in the serum of patients after breast cancer treatment and healthy volunteers.

ICAM-1 and PECAM-1 serum levels in healthy donors were 230 [195; 257] and 67 [62; 78] ng/mL, respectively. In the group of patients following breast cancer treatment, the serum levels of ICAM-1 and PECAM-1 were 555 [511; 659] and 98 [81; 123] ng/mL, respectively. In an intergroup comparison, patients after breast cancer treatment showed a statistically significant increase in the level of ICAM-1 and PECAM-1 molecules (Table 4). For clarity, the results are also demonstrated in Figure 1.

Patients after breast cancer treatment were divided into subgroups according to the following characteristics: the presence of vestibulo-atactic syndrome, depression, polyneuropathy, lymphedema, breast cancer hormone-receptor status, as well as anamnesis of radiation therapy and chemotherapy. The level of adhesion molecules and statistical analysis results are presented in Table 5.

Patients were also divided into subgroups depending on the histological type of breast cancer. The levels of molecules in the subgroups and the results of statistical analysis are presented in Table 6.

The analysis revealed a significant reliable increase in PECAM-1 and ICAM-1 molecules in patients following breast cancer treatment with depression in comparison to patients without depression. Furthermore, a significant increase in the level of PECAM-1 and ICAM-1 molecules in patients with vestibulo-atactic syndrome in comparison to patients without vestibulo-atactic syndrome was reported.

The significant increase in the serum levels of PECAM-1 and ICAM-1 was detected in patients after chemotherapy in comparison to patients without chemotherapy.

The presence or absence of lymphedema did not affect the level of the analyzed molecules, or the presence or absence of polyneuropathy.

No differences in the serum levels of PECAM-1 and ICAM-1 were observed in the groups of patients with or without radiation therapy. Furthermore, there was also no statistically significant difference in the levels of adhesion molecules (ICAM-1, PECAM-1) depending either on hormonal status or the histological type of breast cancer. No statistically significant difference in the age of the patients in the subgroup analysis was found.

## 4. Discussion

Our study revealed numerous signs of CNS damage, manifested in the form of characteristic complaints and psycho–emotional disorders, which coincides with the data of other researchers [21,22]. The use of neuropsychological testing methods, careful assessment of complaints, and neurological status allow us to identify often gross violations of the functions not only of the upper limb on the side of the lesion in breast cancer survivors, but also central and peripheral nervous system damage, violation of the quality of life, psychological disorders, and the formation of anxiety and depressive disorders.

Our study analyzed the levels of soluble adhesion molecules in patients >12 months after radical treatment of breast cancer as potential biomarkers for the prediction of CNS damage. The obtained results, when compared to a group of healthy women, indicated a significant increase in ICAM-1 and PECAM-1 serum levels in patients after radical breast cancer treatment. Indeed, intercellular adhesion molecules are elevated in many diseases (e.g., atherosclerosis, mental illness, chronic inflammatory diseases, etc.). However, the presence of these diseases was an exclusion criterion in the current study protcol, which suggests that in this clinical group, the increase in adhesion molecules might reflect the CNS damage. Inter-cellular adhesion molecules are considered as markers for endothelial cell dysfunction and subclinical inflammation, as a result of microvascular damage [26,27]. The potential role of endothelial damage in reducing cognitive functions after treatment in cancer patients has already been shown in previous studies [28,29]; however, in these studies, the emphasis is placed on the violation of higher psychological functions (i.e., memory, attention, information processing speed), and not on complex damages of the endothelium in the CNS. The direct neurotoxic effect of chemotherapeutic drugs, genetic predisposition to a hyper-inflammatory response to treatment, and oxidative stress were also considered as possible mechanisms of nerve tissue damage [30]. In our work, we assumed that endothelial dysfunction leading to tissue hypoxia plays a central role in all manifestations in patients after the radical treatment of breast cancer. This assumption has been confirmed by studies indicating damage to the vascular wall by chemotherapeutic agents [31,32], radiation therapy [15,33,34] and the direct toxic effect of the tumor [35]. At the same time, in the acute phase, there was an increased permeability of the vascular wall as a result of endothelial cell apoptosis and, as a result, vascular collagen deposition [36] and, in the long-term period, arteriosclerosis, which causes tissue ischemia [37]. We can assume that the same processes also occurs in the CNS in the course of disease, as well as after antitumor treatment in patients with breast cancer. Taking into account the peculiarities of the microvascular structure of the central nervous system, the neurovascular units (neurovascular unit)—groups of closely related cells and components of the extracellular matrix that are involved in the homeostatic and hemodynamic regulation of brain metabolic processes—will be the first to be involved in the pathological process [38,39]. At the same time, inside the neurovascular unit, the vessels are surrounded by glial cells, which serve as an additional barrier in the interaction of the circulatory system and neurons [40]. Thus, it can be assumed that after endothelial dysfunction, first glial cells and then neuronal cells will be affected. Taking into account the role of oligodendrocytes in the synthesis of myelin shells [41], vascular dysfunction will primarily cause axon demyelination and diffuse microstructural damage of the white matter tracts of the brain. This assumption is confirmed by a pronounced decrease in the anisotropy of the white matter tracts detected by diffusion tensor MRI [42,43] and a violation of brain connectivity during fMRI in patients after the complex treatment of breast cancer [44,45,46]. Our data, suggesting that intercellular adhesion molecules are increased especially in the blood of women with neurological deficiency, confirms the assumption that endothelial dysfunction plays a leading role in central nervous system damage. Our study found a statistically significant increase in ICAM-1, PECAM-1 in the blood of women with depression. It can be assumed that depression in women following the treatment is not only situational and functional, but also has a pathomorphological basis as a chronic violation of cerebral circulation after endothelial dysfunction [18,47]. Furthermore, as was shown in the study by Machelska et al. that ICAM-1 expressed on endothelial cells can recruit leukocytes to promote the local control of inflammation, indicating the involvement of this molecule into the pathogenesis of pain syndrome [48].

A statistically significant increase in intercellular adhesion molecules in a subgroup of patients receiving chemotherapy confirms previously published data showing a significant effect of chemotherapeutic drugs on the structure and function of the CNS, including direct toxic effects on glial cells [49,50]. However, it should be noted that high serum levels of ICAM-1 and PECAM-1 were also found in the subgroup of women whose treatment did not include chemotherapy. This result confirms the theory about the complex effect of the tumor and antitumor treatment on the CNS [51,52,53]. Since there were no differences between the subgroup of patients who received radiation therapy, it can be assumed that the radiation itself does not significantly affect the serum levels of ICAM-1 and PECAM-1.

The study also showed an increase in ICAM-1 in a subgroup of women with vestibulo-atactic syndrome. It is possible that this result reflects a pronounced damage of the white matter in these patients, which reduces the functionality of the sensorimotor integrative function of the CNS, which is necessary to maintain postural balance [54].

**Figure 2:**
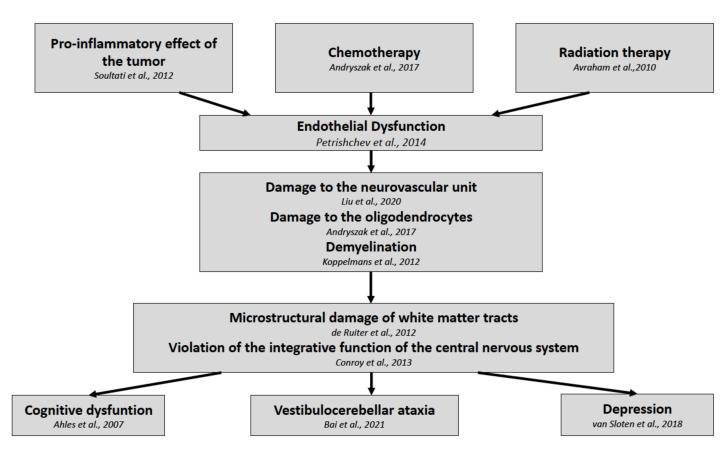
Pathophysiological development of post-treatment symptoms in breast cancer survivors.

According to our results, we can assume that the damage of the white matter tracts of the brain is a result of endothelial dysfunction in patients following the treatment protocol (Figure 2). In favor of this hypothesis is the recently published study by Bukkieva et al., where the authors employing resting state functional magnetic resonance imaging (rs-fMRI) demonstrated the changes in functional connectivity in patients following breast cancer treatment [55]. The levels of soluble ICAM-1 and PECAM-1 could provide biomarkers for predicting the degree of damage in the CNS, and longitudinal studies might enable the monitoring of the effectiveness of therapeutic and rehabilitation measures.

## 5. Conclusions

Development of novel reliable biomarkers to predict CNS complications in women following breast cancer treatment represents one of the trends in clinical oncology. In the current study, we demonstrated that two biomarkers, ICAM-1 and PECAM-1, were significantly higher in the serum of patients associated with cognitive dysfunction, depression, vestibulocerebellar ataxia, and in the group of patients following chemotherapy. We assume that the markers might be predictive of cerebro-vascular damage, which is the underlying basis of treatment complication onset and progression.

## Figures and Tables

**Table 1:** Characteristics of the patients.

Group Characteristics of Patients	Patients after Breast Cancer Treatment n = 45	Healthy n = 25
Age (years)	46.0 [41; 48]	40.0 [36; 44]
Years since treatment	4.5 [3; 7.6]	-
Number of patients TNM stage		
I (T1N0M0)	10	-
II A (T2N1M0)	21	-
II B (T3N1M0)	5	-
III A (T3N2M0)	8	-
III B (T4N2M0)	1	-
Types of breast cancer		-
Ductal carcinoma in situ (DCIS)	8	-
Invasive ductal carcinoma (IDC)	33	-
Invasive lobular carcinoma (ILC)	4	-
Breast cancer hormone receptor status		
Hormone receptor-positive (HR+)	37	-
Hormone receptor-negative (HR−)	8	-
Treatment of breast cancer		
Complex treatment (tumor debulking, radiotherapy, chemotherapy)	20	-
Сombination of surgical treatment and chemotherapy	16	-
Сombination of surgical treatment and radiotherapy	9	-
Hormonal therapy (tamoxifen vs GH-LH analogues)		-
Do not take the medicine	8	-
Take the medicine	25	-
Completed the course	12	-

**Table 2:** Symptoms and complaints in breast cancer survivors.

Complaints and Symptoms	Number of Patients (N, %)
Complaints about edema of the arm, armpit	30 (66%)
Numbness of the hand	26 (57%)
Shoulder blade/chest pain on the side of the operation	20 (44%)
Pain in the arm	31 (69%)
Vertigo	25 (55%)
Back pain	26 (57%)
Unsteadiness when walking	13 (28%)
Anxiety	23 (51%)
Reduced mood background	15 (33%)
Sleep disorders	13 (28%)
Memory decline	23 (51%)
Headache	26 (57%)
Numbness in the distal extremities	26 (57%)
Restriction of movement in the shoulder	15 (33%)
Lymphedema of the arm	30 (66%)
Polyneuropaty	20 (45%)
Vestibulo-atactic syndrome	21 (47%)

**Table 3:** The results of the psychological and functional scales in group of patients after breast cancer treatment.

Indicators	Score
SF-36: overall physical well-being	47.04 [35.11; 58.8]
SF-36: general mental well-being	46.24 [38; 59.2]
EORTC QLQ C30	48.24 [39.5; 52.6]
Zung depression scale	41.5 [38.4; 46.3]
DASH scale	62.97 [56.4; 66.3]

**Table 4:** Adhesion molecules PECAM-1 and ICAM-1 in the serum of patients following breast cancer treatment and healthy volunteers.

Adhesion Molecules	Patients n = 45	Healthy n = 25	Mann–Whitney U Test	Significance (*p*)
PECAM-1, ng/mL	98 [81; 123]	67 [62; 78]	209	<0.001 *
ICAM-1, ng/mL	555 [511; 659]	230 [195; 257]	0	<0.001 *

*—differences between the groups were significant at *p* < 0.05.

**Table 5:** Adhesion molecules PECAM-1 and ICAM-1 in the serum of patients.

Sign of Separation	Presence of the Sign	Number of Patients (and Age)	PECAM-1	Mann–Whitney U Test	*p*	ICAM-1	Mann–Whitney U Test	*p*
Presence of edema	yes	24 (44.3 [40.2; 48])	124 [90; 129]	188.5	0.148	619 [479; 706]	238.0	0.750
	no	21 (41.3 [38.6; 47])	101 [81; 152]			562 [539; 597]		
Depression	yes	18 (39.8 [37.5; 45.4]	131 [105; 166]	137.5	0.014 *	640 [556; 749]	145.5	0.024 *
	no	27 (42.1 [39; 46.1]	78 [90; 112]			528 [478; 577]		
Vestibulo-atactic syndrome	yes	21 (42 [39.4; 47])	132 [120; 162]	135.5	0.008 *	661 [589; 850]	101.5	<0.001 *
	no	24 (41 [38.6; 45.5])	92 [81; 102]			553 [510; 564]		
Radiation therapy	yes	24 (39 [37.4; 44.5])	102 [84; 136]	241.0	0.802	542 [478; 607]	246.0	0.891
	no	21 (42 [38.4; 46.4])	93 [80; 129]			563 [511; 627]		
Chemotherapy	yes	36 (40.6 [38.5; 45.6])	114 [88; 132]	85.5	0.030 *	592 [539; 706]	31.5	<0.001 *
	no	9 (45 [42.4; 49])	81 [78; 91]			453 [447; 483]		
Polyneuropathy	yes	20 (41.5 [38.7; 46])	107 [86; 153]	232.5	0.689	575.5 [536; 659]	219.5	0.486
	no	25 (38 [36.5; 42])	104 [81; 123]			559 [510; 637]		
Breast cancer hormone receptor status	HR+	37 (44 [41; 47])	93 [78; 124]	141.5	0.847	539 [511; 657]	144	0.905
	HR-	8 (42 [39.4; 47])	103 [82; 108]			560 [514; 663]		

*—differences between the groups were significant at *p* < 0.05.

**Table 6:** Levels of adhesion molecules depending on histological type of breast cancer.

Types of Breast Cancer	Number of Patients	PECAM-1	H Kruskal–Wallis	*p*	ICAM-1	H Kruskal–Wallis	*p*
DCIS	8	89 [76; 99]	2.354	0.308	538 [498; 563]	1.870	0.393
IDC	33	101 [82; 127]			563 [512; 678]		
ILC	4	96 [87; 109]			526 [502; 592]		
